# Protein kinase C beta II suppresses colorectal cancer by regulating IGF-1 mediated cell survival

**DOI:** 10.18632/oncotarget.8062

**Published:** 2016-03-14

**Authors:** Catríona M. Dowling, James Phelan, Julia A. Callender, Mary Clare Cathcart, Brian Mehigan, Paul McCormick, Tara Dalton, John C. Coffey, Alexandra C. Newton, Jacintha O'sullivan, Patrick A. Kiely

**Affiliations:** ^1^ Graduate Entry Medical School and Health Research Institute (HRI), University of Limerick, Limerick, Ireland; ^2^ Department of Life Sciences, and Materials and Surface Science Institute, University of Limerick, Limerick, Ireland; ^3^ Stokes Research Institute, University of Limerick, Limerick, Ireland; ^4^ Department of Surgery, Trinity College Dublin, Dublin, Ireland; ^5^ Department of Pharmacology, University of California at San Diego, La Jolla, CA, USA; ^6^ GEMS, St. James Hospital, Dublin, Ireland; ^7^ 4i Centre for Interventions in Infection, Inflammation and Immunity, Graduate Entry Medical School, University of Limerick, Limerick, Ireland

**Keywords:** protein kinase C, IGF-1, cell survival, tumour suppressor, colorectal cancer

## Abstract

Despite extensive efforts, cancer therapies directed at the Protein Kinase C (PKC) family of serine/threonine kinases have failed in clinical trials. These therapies have been directed at inhibiting PKC and have, in some cases, worsened disease outcome. Here we examine colon cancer patients and show not only that PKC Beta II is a tumour suppressor, but patients with low levels of this isozyme have significantly decreased disease free survival. Specifically, analysis of gene expression levels of all PKC genes in matched normal and cancer tissue samples from colon cancer patients revealed a striking down-regulation of the gene coding PKC Beta in the cancer tissue (*n* = 21). Tissue microarray analysis revealed a dramatic down-regulation of PKC Beta II protein levels in both the epithelial and stromal diseased tissue (*n* = 166). Of clinical significance, low levels of the protein in the normal tissue of patients is associated with a low (10%) 10 year survival compared with a much higher (60%) survival in patients with relatively high levels of the protein. Consistent with PKC Beta II levels protecting against colon cancer, overexpression of PKC Beta II in colon cancer cell lines reveals that PKC Beta II reverses transformation in cell based assays. Further to this, activation of PKC Beta II results in a dramatic downregulation of IGF-I-induced AKT, indicating a role for PKCs in regulating IGF-1 mediated cell survival. Thus, PKC Beta II is a tumour suppressor in colon cancer and low levels serve as a predictor for poor survival outcome.

## INTRODUCTION

Following the discovery that the Protein Kinase C (PKC) family members were high affinity intracellular receptors for phorbol-ester tumour promotors, the serine/threonine kinases were studied intensively in the context of targeting them in cancer [1–5]. Yet their precise role in cancer has remained elusive.

PKC is a family of serine/threonine kinases whose 9 members are expressed in many different tissue types and have diverse biological functions. The PKC isozymes are responsible for mediating several biological processes including cell-cycle regulation, cell survival, cell adhesion and the regulation of apoptosis [6, 7]. All members of the PKC family share common basic structures; a flexible hinge segment linking a cell membrane targeting N-terminal regulatory moiety to a C-terminal catalytic domain [8, 9]. The regulatory moiety contains two discrete membrane targeting modules and a pseudosubstrate segment that maintain the enzyme in an inactive conformation [9, 10]. The maturation of PKCs into this autoinhibited conformation is dependent on sequential phosphorylation steps at three highly conserved sites termed the activation loop, the turn motif, and the hydrophobic motif [11–13]. Following these phosphorylation events, the protein is activated by specific secondary messengers that bind to the regulatory domain to mediate the release of the pseudosubstrate segment from the active site [10, 14]. The classical PKCs are targeted by diaclyglycerol (DAG) and Ca^2+^, the novel PKCs are activated by DAG alone, and the atypical PKCs bind neither DAG nor Ca^2+^, instead are regulated by protein:protein interactions [6, 15]. Prolonged activation of PKCs with phorbol esters results in a downregulation of the protein as a result of dephosphorylation and subsequent degradation [16–18].

Research into the role of different PKC isoforms in cancer is based primarily on the assumption that increased PKC activation and expression promotes carcinogen induced tumorigenesis [1–3, 19, 20]. A large body of evidence supports this, for example, overexpression of PKCε is a suggested tumour promoter in stomach, lung, thyroid, colon and breast cancer [4], increased levels of PKCη are associated with tumour aggressiveness and an increased rate of cell proliferation in non-small-cell lung cancer [21] and overexpression of PKC θ is linked to gastrointestinal stromal tumours [22]. However, immunohistochemical and biochemical studies indicate that altered expression of the PKC isoforms is variable and depends on the cancer type [5, 23, 24]. For example, it has been shown that PKCα can both induce and supress colon cancer cell proliferation [25, 26]. Similarly, PKCζ overexpression in colon cancer cell lines decreases tumour formation in nude mice while loss of PKCζ is also associated with decreased tumorigenicity [27, 28]. The expression of PKCβ in breast, gastric and colon cancer has been subject to much debate and there are many studies presenting arguments for both up and down regulation of the isoform in cancer cells and cancer tissue [29–34].

Collectively, this research has driven the development of several target therapies against cancer [5, 34, 35]. These targeted therapies are showing promise as potential drugs in the treatment of cardiac diseases [36–40] and bipolar mania [41–43], suggesting that there is merit in targeting PKC [5]. However, PKC inhibitors have proved unsuccessful as anti-cancer agents in clinical trials [5, 44]. Further to this, meta-analysis conducted on controlled clinical trials of chemotherapy alone versus chemotherapy combined with PKC inhibitors discovered response rates and disease control rates were significantly reduced in the groups that were administrated PKC inhibitors [45]. Taken together, this challenges the view that PKCs are acting as tumour promotors and strongly suggests that a recalibration of our understanding of how PKC's function in cancer may be required. In fact, recent work examining PKC mutations in human cancers revealed that approximately 60% of PKC mutations actually reduced or abolished PKC activity and none were activating, proposing that PKCs could be playing a tumour suppressor role [46]. This study also demonstrated that correction of one loss of function PKC Beta II mutation in a colon cancer cell line actually decreased tumour size in mouse xenografts [46].

In our investigation, we set out to compare the expression of all the PKC isoforms in colorectal cancer to understand whether its function was enhanced or reduced in this cancer. Keeping in mind that PKCs are differentially expressed across and within different tissues, our approach was to reduce any variability that may occur between patients by examining the expression of the genes encoding PKCs in patients matched normal and colorectal cancer tissue. Our analysis revealed a dysregulation of many of the isoforms with a striking down regulation of the PKC Beta II isoform in the cancer tissue. To further investigate the implication of this in colorectal cancer, we examined the protein expression of PKC Beta II across a large cohort of patients and compared expression of PKC Beta II in normal distant tissue, normal adjacent tissue, cancer tissue and tumour edge tissue. Interestingly, this revealed a dramatic downregulation of the protein in the cancer and tumour edge tissue. Further to this, we found a strong association between low levels of PKC Beta II in normal distant tissue and a reduction in disease free survival time for colorectal cancer (CRC) patients. To substantiate our findings, we overexpressed PKC Beta II in colon cancer cells and used a series of cell based assays including real-time cell migration and cell invasion assays to reveal that overexpression of PKC Beta II reverses the transformed phenotype. Upon further investigation we revealed that both activation and overexpression of PKC Beta II dramatically reduced phosphorylation of AKT and consequently reduced cell survival. All these data combined strongly suggest that PKC Beta II has a tumour suppressor role in colon cancer.

## RESULTS

### The genes encoding protein kinase C isoforms are dysregulated in colon cancer

**Figure 1:**
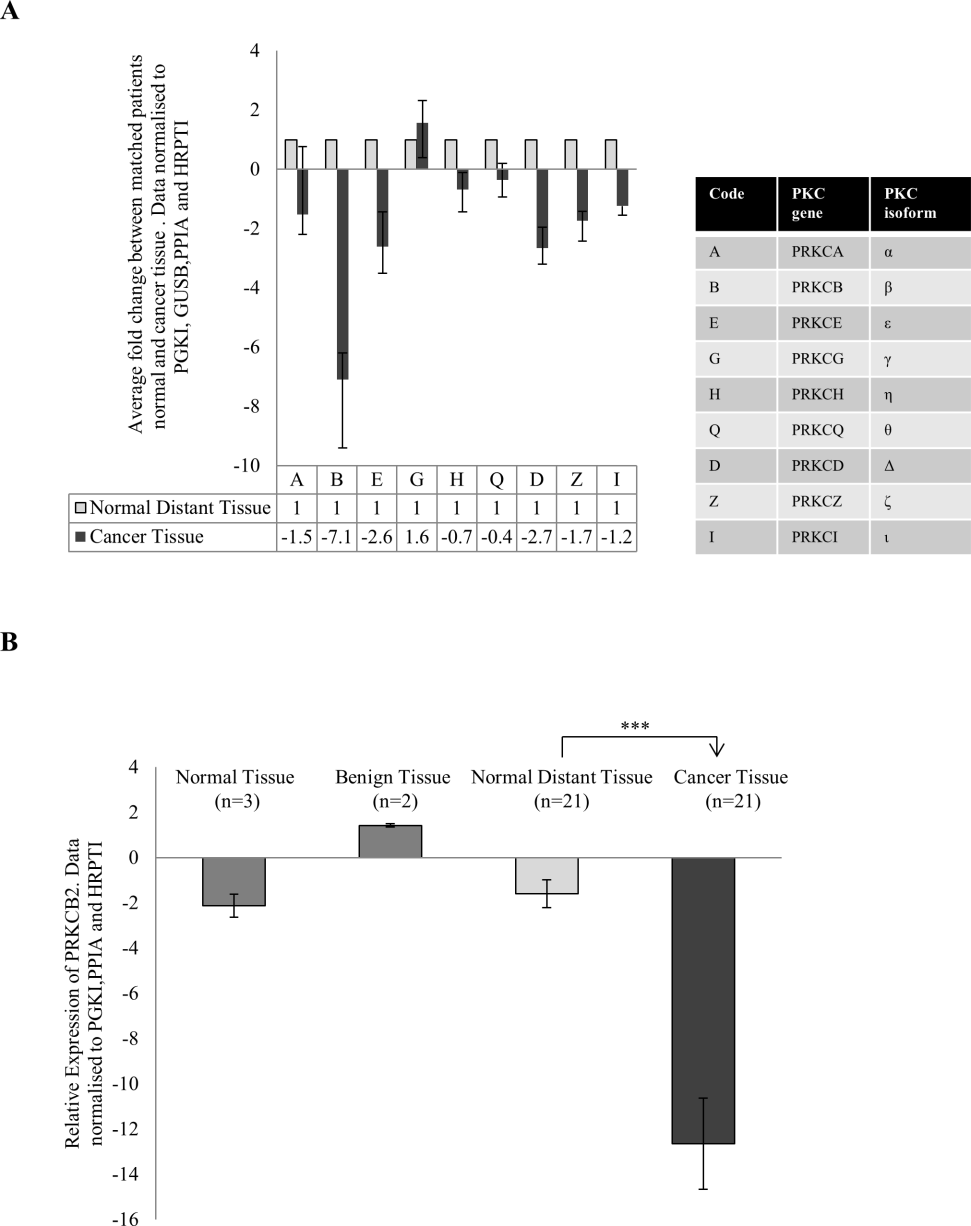
Gene expression of PKC genes in colon cancer Tissue samples measuring approximately 0.5 cm in diameter were collected from 21 patients undergoing surgery in University Hospital Limerick. Normal tissue from the 21 patients was also collected approximately 10 cm away from the cancer tissue. RNA was extracted from the tissue, cDNA was synthesised and real time PCR was carried out. All data was normalized using the housekeeping genes PRGK1, GUSB, PPIA and HRPT1. (**A**) Average fold change of the 9 PKC coding genes in cancer tissue of patients compared to each patients normal tissue (*n* = 21). Each individual patients fold change was analysed using the REST^©^ software. The table explains the corresponding PKC coding gene and PKC isoform. (**B**) Gene expression of PRKCB in normal tissue (*n* = 3), benign tissue (*n* = 2) and cancer tissue (*n* = 21) relative to normal distant tissue (*n* = 21). Data was analysed using the delta delta ct method (Statistical significance based on the Welch test and Bonferroni test ***p < 0.01).

To investigate the change in PKC expression in colon cancer, we used fresh tissue samples that were excised from both the cancer tissue and normal distant tissue of individual patients. Using real time PCR, we compared PKC expression in their normal distant tissue to their cancer tissue and established an individual fold change for each patient. After averaging the fold change of all patients for each PKC coding gene, PRKCB, PRKCD and PRKCE demonstrated a down-regulation greater than two while PRKCG was upregulated (Figure 1A, S1). This suggests that there is differential expression of the genes encoding PKC when the cancer tissue and normal tissue of individuals are compared. The most striking dysregulation was the downregulation of the gene coding for the protein PKC Beta II. This dysregulation was not influenced by the progression of the disease as no statistical difference was found between stage 2 and stage 3 patients (Supplementary 2A). To investigate this further we examined the relative expression of PRKCB in normal distant tissue and compared it to cancer tissue, normal tissue and benign tissue (Figure 1B, S2B). We found a significant downregulation of PRKCB in the cancer tissue compared to the normal distant tissue (*p* < 0.01), with no difference between the other tissue groups examined.

### PKC Beta II is down-regulated in tumours from CRC patients

**Figure 2:**
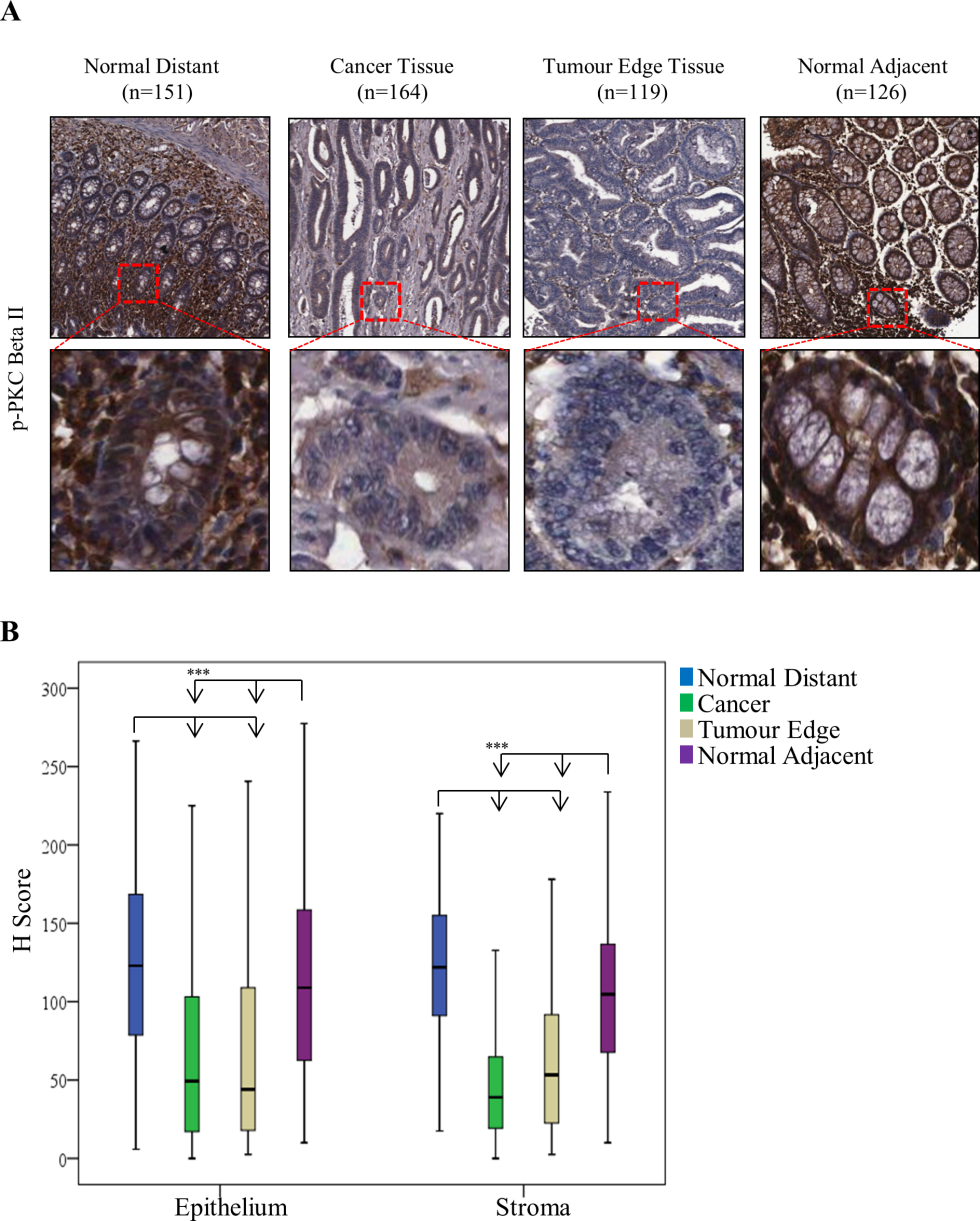
Expression of PKC Beta II in CRC patients PKC Beta II expression was assessed by immunohistochemistry in normal distant, cancer, tumor edge and normal adjacent tissue samples from CRC patients (*n* = 166). (**A**) Representative image of PKC Beta II staining for Normal Distant (*n* = 151), Cancer (*n* = 164), Tumor Edge (*n* = 119) and Normal Adjacent (*n* = 126) tissue. (Top panel images = 5X, Bottom panel images = 20X). (**B**) Box plot representing the difference in PKC Beta II H score between normal distant, cancer, tumor edge and normal adjacent in epithelium (*n* = 151, 164, 119, 126) and stromal tissue (*n* = 152, 166, 121, 126) (Statistical difference based on Welch Test and Bonferroni test, ***p < 0.001).

The changes we observed in the expression of PKC Beta II are noteworthy as there are some conflicting results in the literature. Earlier studies suggested that PKC Beta II expression is upregulated and is an early event in colon cancer in mice [32]. This considered we next wanted to determine if this downregulation was observed at a protein level in CRC patients. To do this, we examined the expression of mature PKC Beta II in 197 CRC tissue samples (Table 1), using tissue microarrays and immunohistochemistry. As expected, and in support of our gene analysis, expression of PKC Beta II was significantly reduced in the cancer epithelium tissue when compared with normal distant epithelium tissue (*p* < 0.01) (Figure 2A, 2B). Similar results were found when comparing cancer stromal tissue with normal distant stromal tissue (*p* < 0.01) (Figure 2A, 2B).

**Table 1:** Characteristics of the cohort of colorectal cancer (CRC) patients and summary of the pathology of the tumour microarrays used to analysis the expression of PKC Beta II

	Stage 1	Stage 2	Stage 3	Stage 4
**N**	7	24	161	5
**Male/Female**	5/2	7/17	96/65	1/4
**Age, median (range)**	69 (45–75)	65 (45–86)	72 (34–94)	67 (53–92)
**CRC mortality**	0	8	105	4
**Lymphvascular Invasion**	0	2	136	0
**Metastatic**	0	6	24	5

In addition, we examined the expression of mature PKC Beta II in tumour cores representative of normal adjacent and tumour edge tissue. Again, expression was significantly reduced in the tumour edge epithelium tissue (*p* < 0.01) when compared with normal distant epithelium and normal adjacent epithelium tissue, while no significant difference was observed between tumour edge epithelium and cancer epithelium tissue (Figure 2A, 2B). Similar results were also obtained for the stromal tissue (Figure 2A, 2B). Comparable to our gene expression analysis, progression of the disease did not influence the protein levels of PKC Beta II (Supplementary 3). To the best of our knowledge, our data show for the first time that PKC Beta II is down-regulated at both the gene and protein level in CRC tissue.

### Low expression of PKC Beta II in normal distant epithelium tissue is associated with a reduction in disease free survival time

To test whether a low expression of PKC Beta II was associated with a poor prognosis we compared the survival of patients with low and high expression of PKC Beta II. Low and high expression values were determined using histograms; with values less than the median designated low expression, and those greater than the median designated high expression (Supplementary 4A, 4B).

**Figure 3:**
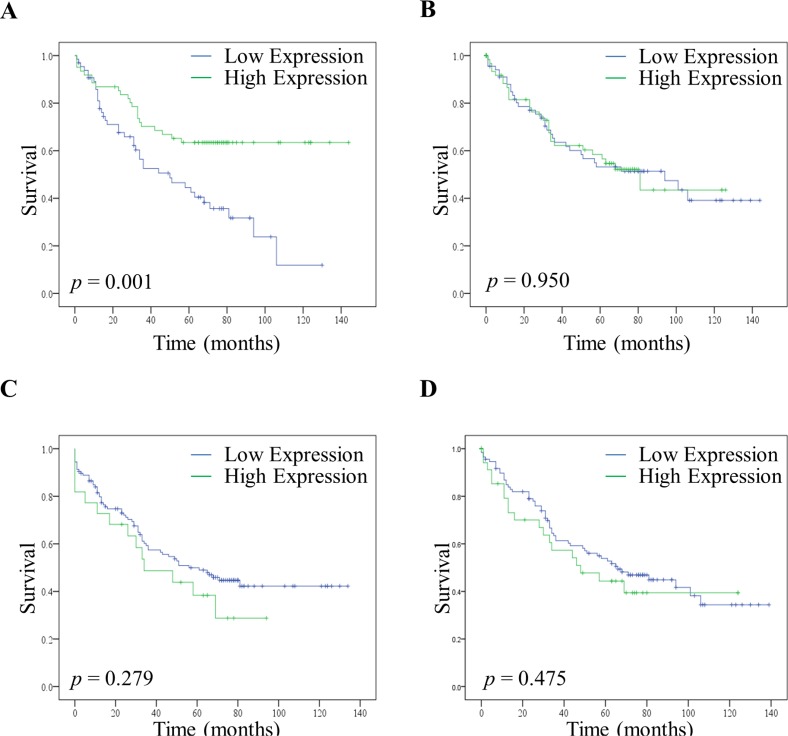
Disease free survival of patients with high and low expression of PKC Beta II High and low expression of PKC Beta II was determined using the median H score value for normal distant tissue and cancer tissue. (**A**) Effect of high (*n* = 61) and low (*n* = 65) expression of PKC Beta II on disease free survival in the normal distant epithelium tissue of CRC patients. (**B**) Effect of high (*n* = 75) and low (*n* = 77) expression of PKC Beta II on disease free survival in the normal distant stromal tissue of CRC patients. (**C**) Effect of high (*n* = 22) and low (*n* = 126) expression of PKC Beta II on disease free survival in the cancer epithelium tissue of CRC patients. (**D**) Effect of high (*n* = 40) and low (*n* = 126) expression of PKC Beta II on disease free survival in the cancer stromal tissue of CRC patients. *P*-values represent the Log-rank test.

Remarkably, although fewer patients had low expression of PKC Beta II in their normal distant tissue (Supplementary 4C, 4D), those patients that did have low expression in their normal distant epithelium tissue showed a significant reduction in their disease free survival time (*p* < 0.01) (Figure 3A). Interestingly, this was only true for the normal distant epithelium tissue as no significant difference was observed between high and low expression in the normal distant stromal tissue (Figure 3B). Moreover, there was no reduction in the disease free survival time associated with low and high expression in either the cancer epithelial tissue (Figure 3C) or the cancer stromal tissue (Figure 3D). This reflects our findings that the downregulation of PKC Beta II is not progressive across tumour stage (Figure S2A and S3). Consequently, as expected, lower expression in the cancer epithelium is not associated with a reduction in disease free survival.

### Overexpression of PKC Beta II reverses the transformed phenotype of HCT116 cells

**Figure 4:**
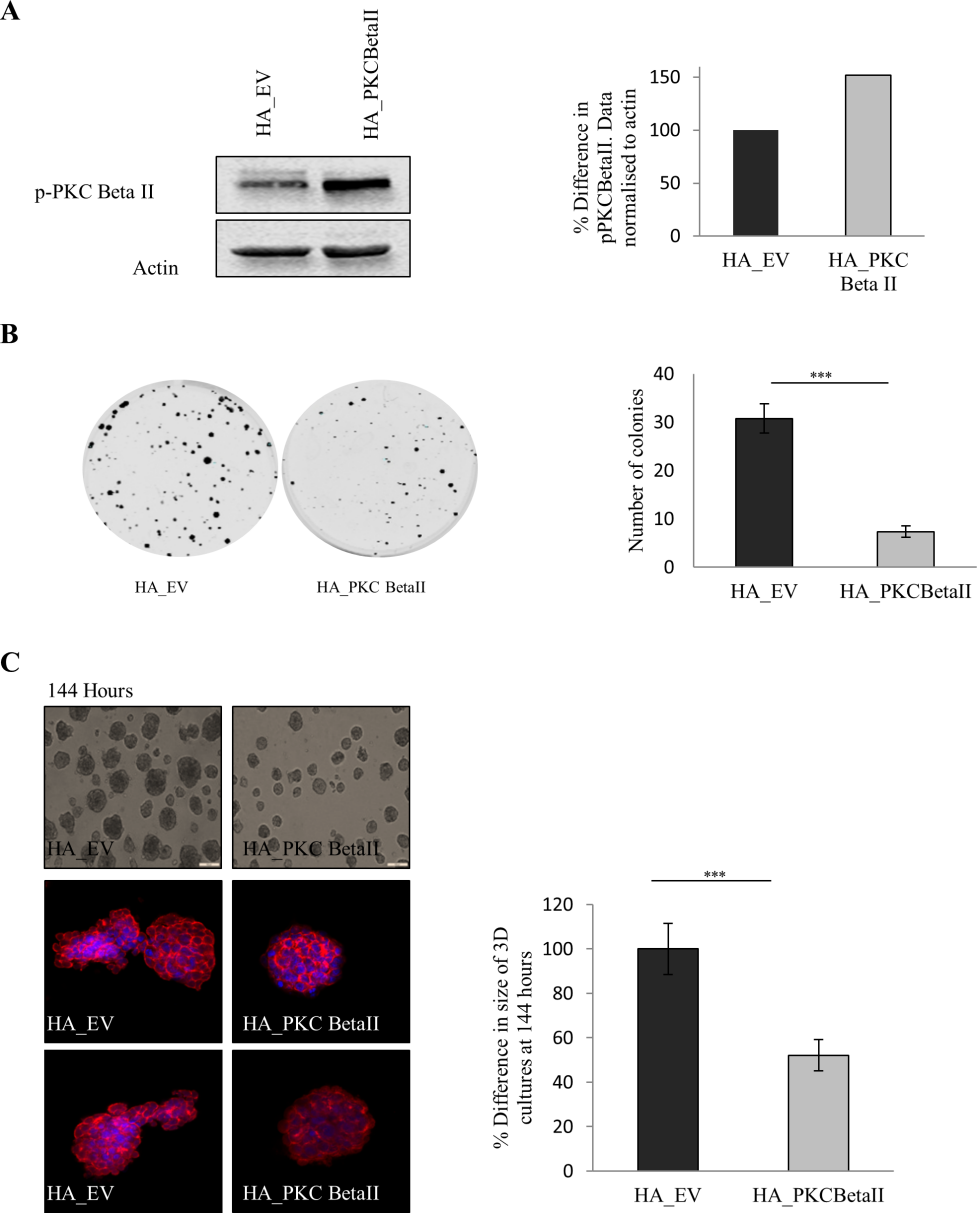

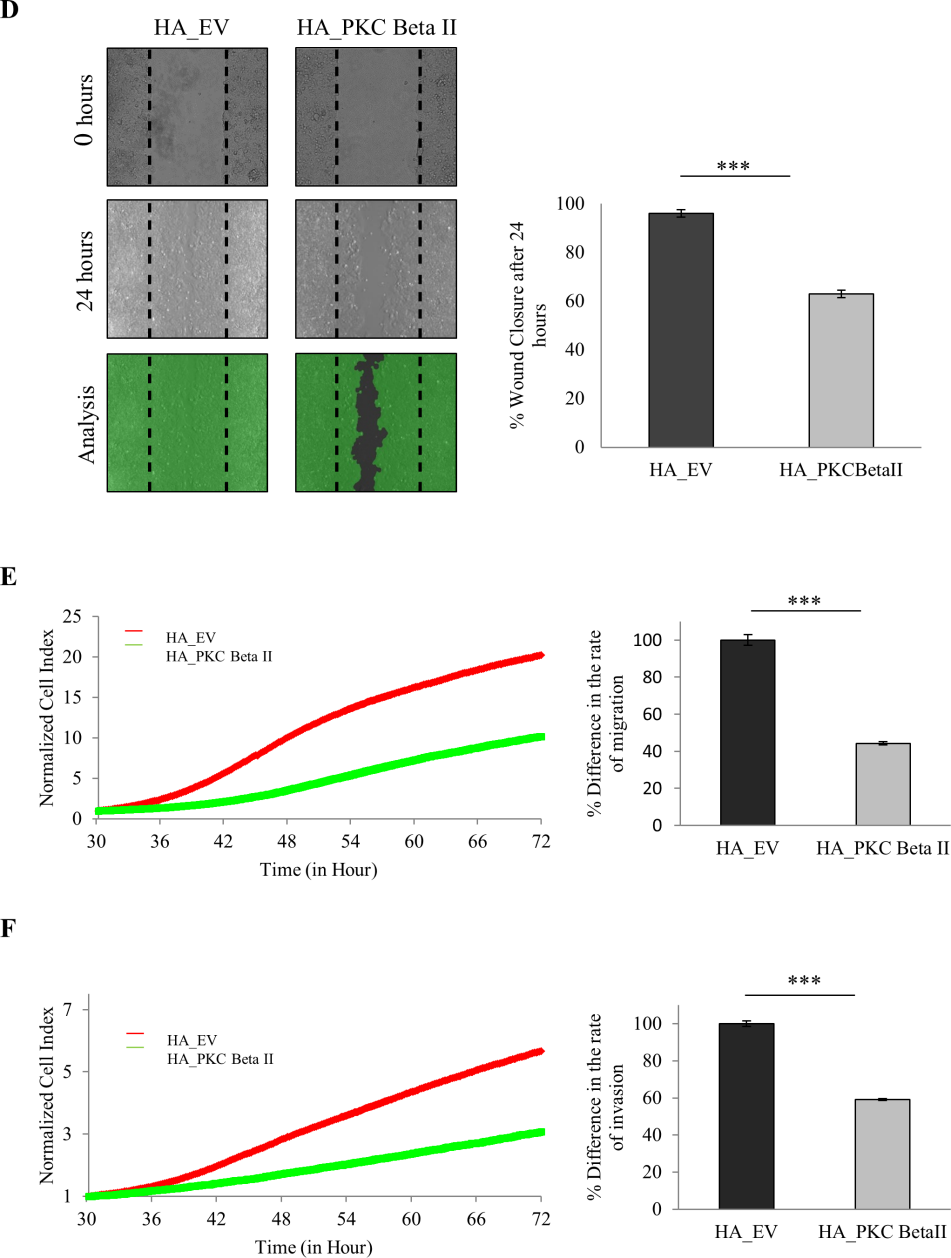
Effect of overexpression of PKC Beta II on colon cancer cells The effect of overexpressing PKC Beta II in colon cancer cells was examined using HCT116 cells stably over expressing HA_PKCBeta II. (**A**) Representative western blot showing the expression of p-PKC Beta II in stable overexpressing HCT116 cells (*n* = 3). (**B**) Representative images demonstrating the difference in the cells ability to form colonies when overexpressing PKC Beta II (*n* = 3). (**C**) Images demonstrating the difference in the size of cells growing in a 3D matrix over 144 hours (*n* = 3). Bar graph represents the percentage difference in the size of 3D cultures at 144 hours. Confocal images are cells harvested and stained after 144 hours (*n* = 3). (**D**) Representative image of cells that were scored with a wound and allowed to migrate over 24 hours. Bar graph represents the percentage wound closer after 24 hours (*n* = 3). (**E**) Representative graph of cells migrating over a period of 72 hours analysed in real time on the xCELLigence system (*n* = 3). Bar graph represents the percentage difference in cell index. (**F**) Representative graph of cells invading over a period of 72 hours analysed in real time on the xCELLigence system (*n* = 3). Bar graph represents the percentage difference in cell index.

Our data indicate that there is a downregulation of PKC Beta II in colorectal cancer. Given this observation we predict that overexpressing PKC Beta II in colon cancer cells would reverse the transformed phenotype. To test this, we generated HCT116 cells overexpressing PKC Beta II (Figure 4A) and carried out a number of cell based assays. Using a colony formation assays, we observed that cells overexpressing PKC Beta II show a significant reduction in the ability to form colonies (*p* < 0.05) (Figure 4B). Next, we wanted to examine if cells overexpressing PKC Beta II would show altered behaviour in 3-Dimensional culture. Strikingly, 3D cultures overexpressing PKC Beta II showed a 50% reduction in culture size after 144 hours (Figure 4C, S5A). In an attempt to get a more comprehensive view of the phenotype of the 3D cultures, they were extracted at 144 hours, fixed, stained and observed using confocal microscopy. Cultures expressing empty vector demonstrated a migratory phenotype, reminiscent of wild type HCT116 cultures, however, in cells overexpressing PKC Beta II, the cultures were more organised and remained small and rounded (Figure 4C).

Next, we tested whether PKC Beta II overexpression would have an influence on the migratory capacity of HCT116 cells. To do this, we carried out a wound migration assay using ibidi^®^ inserts and observed a significant reduction in the ability of cells overexpressing PKC Beta II to migrate into the wound (*p* < 0.01) (Figure 4D). Our observations are due to the decreased migratory behaviour of the overexpressing cells as no difference was observed in the rate of proliferation (Supplementary 5B). Following this, we wanted to monitor the migratory behaviour of the cells in real time using the xCELLigence CIM-plates configuration. Serum starved overexpressing cells were placed in the upper chamber of the CIM-plates with 10% FBS acting as a chemoattractant in the lower chamber. Strikingly, cells overexpressing PKC Beta II showed an 80% reduction in the ability to migrate towards the chemoattractant after 72 hours (*p* < 0.01) (Figure 4E). To test if the invasive property of HCT116 cells was altered in the overexpressing cells, a similar assay was set up, with an additional matrigel layer added to the upper chamber of the CIM-plates to stimulate the ECM and to provide a barrier for the cells to invade [47]. As above, cells overexpressing PKC Beta II demonstrated a significant reduction in the rate of invasion towards the chemoattractant (*p* < 0.01) (Figure 4F). All this information considered would indicate that PKC Beta II plays an important role in maintaining the malignant phenotype of HCT116 carcinoma cells as overexpression of PKC Beta II results in the reversal of this phenotype.

### Overexpression and activation of PKC Beta II reduces cell survival and IGF-1 mediated phosphorylation of AKT

**Figure 5:**
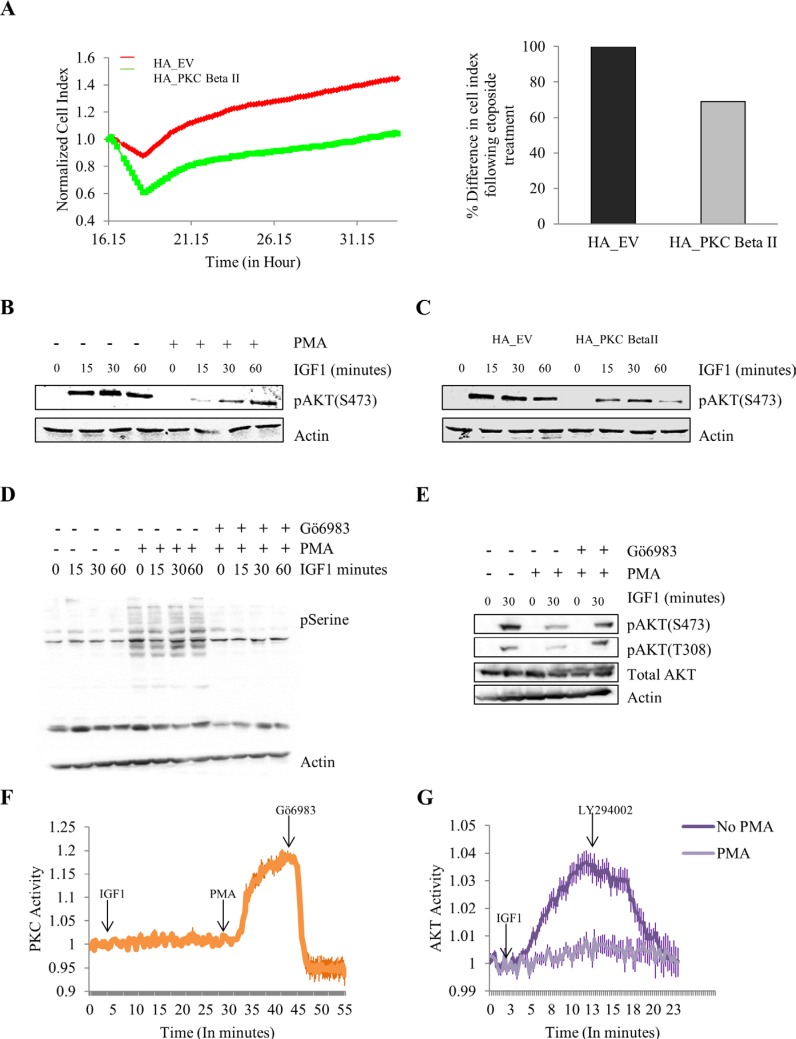
Effect of overexpressing and activating PKC Beta II on protection from cell death and IGF-1 mediated AKT phosphorylation The effect of overexpressing PKC Beta II on protection from etoposide was examined using HCT116 cells stably overexpressing HA_PKCBeta II. Phorbol 12-myristate 13-acetate (PMA) was utilized to activate PKCs following which IGF-1 mediated AKT phosphorylation was examined. (**A**) Representative graph of cells proliferating following etoposide treatment at 16 hours. Data analysed in real time on the xCELLigence system (*n* = 3). Bar graph represents the percentage difference in cell index. (**B**) Representative western blot showing the effect of PMA treatment on the IGF-1 mediated expression of pAKT in HCT116 cells (*n* = 3). (**C**) Representative western blot indicating the difference in IGF-1 mediated phosphorylation of AKT when HCT116 cells are stably overexpressing PKC Beta II (*n* = 3). (**D**) Representative western blot showing the activation of PKC with the addition of PMA and the inhibition of PKC with the addition of Gö6983 (*n* = 3). (**E**) Representative western blot showing the effect of a PKC inhibitor on the PMA induced downregulation of pAKT (*n* = 3). (**F**) Normalised FRET ratio changes (mean +/− SEM) showing PKC activity from HCT116 cells co-expressing CKAR and RFP-tagged PKC Beta II treated with IGF-1, PMA and Gö6983. (**G**) Normalised FRET ratio changes (mean +/− SEM) showing AKT activity from HCT116 cells expressing plasma membrane targeted BKAR treated with IGF-1 and LY294002.

All these results combined indicate that PKC Beta II is acting as a tumour suppressor in colon cancer. To gain an insight into the mechanistic role PKC Beta II plays, we examined the effect of PKC Beta II on the survival pathway in HCT116 cells using a number of different approaches. Firstly, using the xCELLigence system we observed that etoposide induced an increase in cell death in cells stably overexpressing PKC Beta II when compared to empty vector (*p* < 0.01) (Figure 5A). This result indicates that PKC Beta II plays a role in reducing the survival pathway in colon cancer cells. To investigate this further we examined the effect PKC activation had on the IGF-1 induced phosphorylation of AKT. HCT116 cells were treated with PMA or DMSO for 15 minutes prior to IGF-1 at different time intervals. Interestingly, cells pretreated with PMA showed a dramatic downregulation of IGF-1 induced phosphorylation of AKT (Figure 5B). This observation led us to predict that HCT116 cells stably overexpressing PKC Beta II would show reduced phosphorylated AKT compared to empty vector cells. To examine this we treated both groups of cells with IGF- 1, and observed a marked decrease in pAKT expression in the HCT116 cells stably overexpressing PKC Beta II (Figure 5C). To confirm this decrease in pAKT was induced by PKC activation, we next treated cells with the PKC inhibitor, Gö6983, five minutes prior to PMA treatment. As predicted, we observed that the PMA effect was abolished when cells were pretreated with Gö6983 (Figure 5D, 5E). To distinguish the effect IGF- 1 and PMA had on PKC activity we utilized the FRET-based PKC reporter (CKAR), this reporter specifically uses changes in FRET to quantify PKC phosphorylation activity in real-time and in live cells. The phosphorylation-dependent FRET ratio changes revealed that IGF-1 had no effect on PKC activity and the activity was specifically induced by PMA and inhibited by Gö6983 (Figure 5F). Next, using an AKT-specific FRET reporter (BKAR) we wanted to compare the effect of PMA treatment on IGF-1 induced activity of AKT. Strikingly, we observed that PMA treatment showed a dramatic reduction in the phosphorylation activity of AKT compared to untreated HCT116 cells (Figure 5G). The downregulation of PKC Beta II at both a gene and protein level in a large cohort of patients, combined with the reversal of the malignant phenotype of colon cancer cells and the dramatic effect it has on reducing the survival pathway in colon cancer cells, indicates that PKC Beta II is in fact functioning as a tumour suppressor. Together, these data demonstrate that in tumour cells, loss of PKC Beta II is an early event in cancer progression which leads to increased survival and maintenance of the transformed phenotype.

## DISCUSSION

Using matched normal and cancer tissue from CRC patients we have shown dysregulation of PKC coding genes in colorectal cancer patients with the most striking difference being a downregulation of PKC Beta II. Our findings also show that PKC Beta II is dramatically down-regulated at the protein level in the cancer tissue of a large cohort of CRC patients and most interestingly, low expression of the protein in normal distant tissue is associated with a reduction in disease free survival. Further to this, overexpression of PKC Beta II radically decreases cell migration and invasion while also reducing cell survival in colon cancer cells. This study challenges traditional views that PKC isoforms act as tumour promoters, and instead demonstrates that PKC Beta II acts as a tumour suppressor.

Our comprehensive gene expression analysis of all PKC isoforms revealed that three of the nine PKC coding genes (PRKCB, PRKCD and PRKCE) showed a down-regulation greater than two in the cancer tissue, while only one of the genes (PRKCG) was up-regulated. It is also noteworthy that all other isoforms were trending towards down-regulation in the cancer tissue. This somewhat equates to a recent study which demonstrated that 61% of PKC mutations characterized in cancer were loss of function and none were activating [46], suggesting that suppression of PKC activity is a key part of the transformed phenotype. Although the gap in knowledge between expression at the gene and protein level needs to be refined, together, both these findings suggest that PKCs have a tumour suppressor role in colorectal cancer tissue.

We next looked at PKC Beta II expression at the protein level using a large number of cancer and normal tissue samples obtained from CRC patients (Table 1). We found that there was a striking down-regulation of the mature protein in tissue taken from both the tumour itself and tissue taken from the tumour edge. Interestingly also, this reduction in levels of PKC Beta II was observed in both the epithelial and stromal tissue suggesting that PKC Beta II down-regulation across the entire tumour is an important feature of colorectal cancer. This is valuable information as it is becoming more apparent that the stromal environment is an integral part of cancer initiation and progression [48].

One of the most intriguing findings of our study was the strong association found between low levels of PKC Beta II in normal distant tissue and a reduction in disease free survival time for CRC patients. Again, this is evidence that loss of PKC activity is an important and early step in cell transformation. Recent meta-analysis conducted on clinical trials of non-small cell lung cancer patients given chemotherapy alone versus chemotherapy combined with various PKC inhibitors revealed response rates and disease control rates were significantly reduced in the groups that were administrated PKC inhibitors [45]. Interestingly, also, Bryostatin-1, a PKC activator failed in clinical trial in cervical cancer patients, however, this is likely to due to the effects this compound has on down-regulating PKCs [49–51]. As PKC inhibitors are not targeting cancer tissue specifically, it is likely that they are down-regulating PKCs globally in the patient's colonic epithelium. Our findings that CRC patients that naturally exhibit low levels of PKC Beta II in their normal tissue have reduced disease free survival time, and this may explain why patients receiving PKC inhibitors have reduced disease control rates. It is not surprising that no relationship was found between levels of PKC Beta II in cancer tissue and disease free survival time, as we clearly found that the down-regulation of the protein is not enhanced by disease progression.

Considering the data, what effect might an increase in PKC Beta II levels have on colon cancer cells? In agreement with our proposed hypothesis that PKC Beta II inhibits tumour growth, colon cancer cells overexpressing the protein showed a dramatic decrease in the ability to form colonies in plating efficiency assays. The migratory and invasive capacities of these cells, as monitored by real-time cell analysis were also severely compromised. This is supported by previous work in which overexpressing PKC Beta II in colon cancer cells inhibited colony formation in soft agar and correction of a PKC Beta II mutation that restored activity in colon cancer cells reduced tumour size in nude mice [46]. Moreover, when we overexpressed PKC Beta II in colon cancer cells there was a marked reduction in the size of their 3-dimensional cultures and a dramatic change in the migratory phenotype of the cultures. Taken together, this demonstrates that increasing levels of PKC Beta II in colon cancer cells reverses the malignant phenotype of the cells and further points to a tumour suppressor role for PKC Beta II.

**Figure 6:**
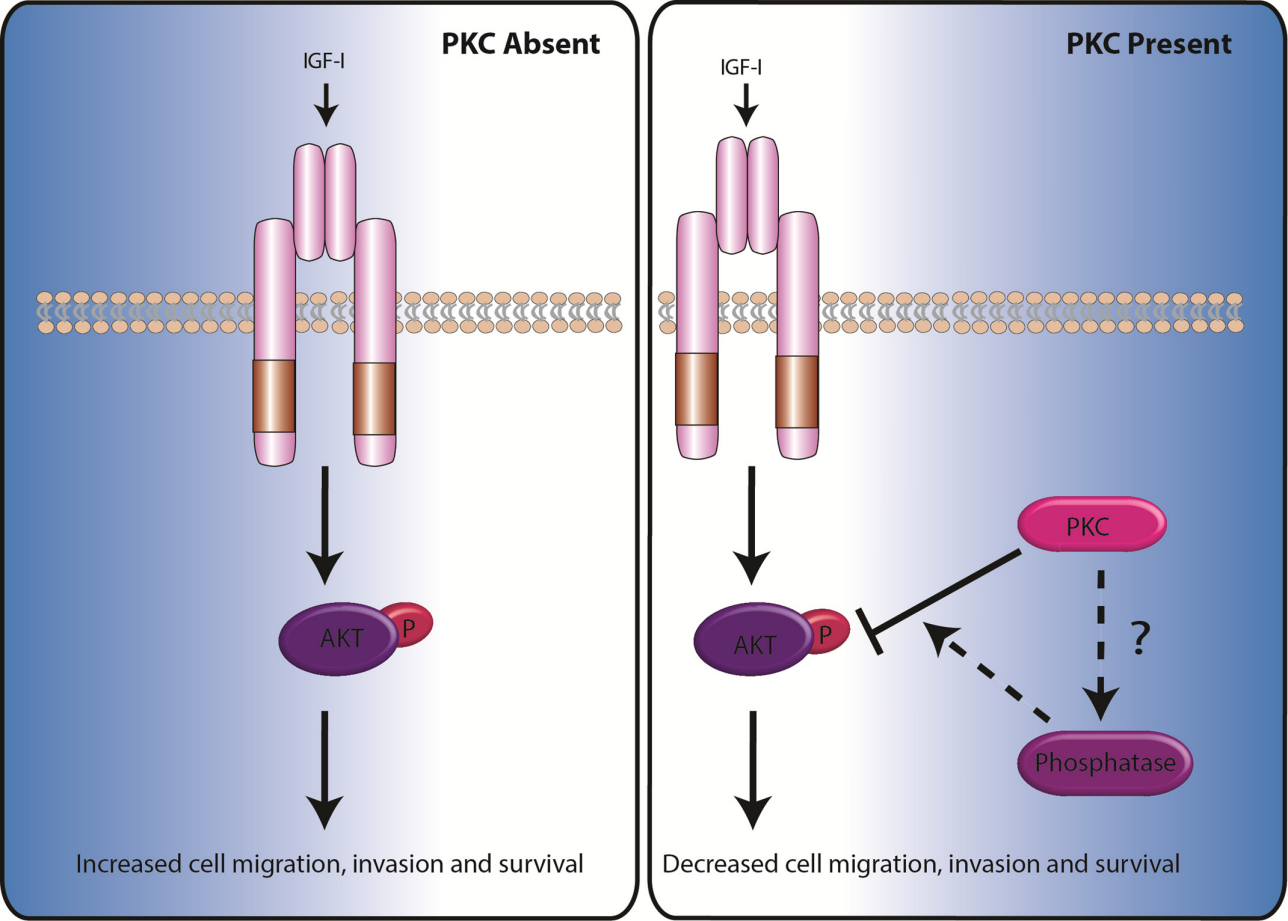
Model demonstrating how the absence or presence of PKC determines the rate of cell migration, invasion and survival For additional information on the signals required to activate PKCs see *Dowling et al. (2015)* [51].

What mechanistic role does PKC Beta II exhibit in order to suppress this cancer phenotype? A common advantageous hallmark of cancer cells is upregulated survival signalling and a resistance to cell death [52]. Considering that the loss of PKC Beta II appears to be an important early step in the formation of the transformed phenotype, we anticipated that PKC Beta II may play an important role in inhibiting the survival pathway. Consistent with this we found that cells overexpressing PKC Beta II showed a reduction in their resistance to cell death induced by the common cytotoxic anti-cancer drug, etoposide. A common mechanism for the promotion of cell survival in cancer is through AKT [53]. In agreement with this we observed a marked downregulation of IGF- 1 mediated phosphorylation of AKT when PKCs were activated in colon cancer cells and in cells overexpressing PKC Beta II. Accordingly, this reduction of AKT phosphorylation was reversed when the cells were treated with a PKC inhibitor. We observed that activation of PKC by PMA was independent of IGF-1 and IGF-1 mediated activation of AKT was significantly reduced in the presence of PMA. These data would strongly suggest that PKC Beta II plays an important role in suppressing the survival pathway in cancer cells making the downregulation of PKC Beta II an imperative early step in the establishment and maintainence of the transformed phenotype (Figure 6).

In this study, our patient analysis combined with our *in vivo* assays provides a strong supporting argument that PKC Beta II functions as a tumour suppressor in the prevention of colorectal cancer. The study is one of the first suggesting this, and is supported by recent analysis of cancer-associated mutations in PKC that revealed that all PKC mutations found in cancer are loss-of-function [46]. This discovery together with our finding that a low level of PKC in normal tissue dramatically reduces disease free survival time would indicate that PKC activation does not precede its downregulation. By looking specifically at normal and cancer tissue from CRC patients, our work has strongly enhanced the hypothesis that PKC Beta II is acting as a tumour suppressor in CRC. Our findings that PKC Beta II is dramatically downregulated at stage one of the disease would suggest that loss of PKC activity is a very early event in the disease. This contrasts with previous work suggesting the up-regulation of PKC Beta II is an early event in colon cancer in mice [32]. These contradictory results reflect a recent commentary in which it was suggested that one of our biggest challenges in targeting PKCs is the limited data that are available on patient samples, and, the difficulties associated with translating animal models in the clinic [44]. Taken together; our findings that PKC Beta II is reduced in the stromal and epithelial tissue of the tumours and that low expression of PKC Beta II in normal tissue of CRC patients dramatically reduces disease free survival time, points strongly towards the idea that a ‘global’ down-regulation of PKC Beta II is an important driver of the disease. This provides a good explanation as to why clinical trials with PKC inhibitors have been largely unsuccessful and some in fact reduced disease control rates. Interestingly, very recent findings have revealed that interrupting K-Ras-calmodulin binding with prostratin, an orally active protein kinase C activator actually represses tumorigenesis in K-Ras driven cancers [54].

These data combined with our *in vivo* assays strongly highlight that PKC Beta II has an important role to play in the suppression of cancer. PKC Beta II may in fact be acting to control the activity of phosphatases which subsequentially regulate AKT and reduces cell survival (see summary Figure 6). All this evidence certainly challenges our current view of PKCs suggesting perhaps we need to reconsider how we target PKCs in the clinic.

## MATERIALS AND METHODS

### Clinical samples

Following ethical approval tissue samples measuring approximately 0.5cm in diameter were collected from 21 patients (median age 68 y; range, 45–84; male 13, female 8) undergoing surgery in University Hospital Limerick. One patient had stage 1, 14 patients had stage 2 and 6 patients had stage 3 colorectal cancer. Normal tissue from the 21 patients was also collected approximately 10 cm away from the cancer tissue. Specimens were immediately placed in Allprotect tissue reagent (Qiagen) and stored at −80°C.

A second cohort of tissue samples was collected, with ethical approval, from 197 patients (median age, 71 y; range, 34–94; male 109, female 88) undergoing surgery at St. James Hospital Dublin. Seven patients had stage 1, 24 patients had stage 2, 161 patients had stage 3 and 5 patients had stage 4 colorectal cancer. Samples were collected from the tumour, tumour edge, normal distant tissue and normal adjacent tissue. After surgery, samples were fixed in 10% formalin and embedded in paraffin.

### RNA extraction and cDNA synthesis

Frozen tissue was immersed in liquid nitrogen and ground into powder. Lysis buffer was added to tissue and the sample transferred to tubes using a 21-gauge needle. Total RNA was extracted as per Qiagen RNeasy Mini Kit instructions. RNA was quantified using a Nanodrop Spectrophotometer (Thermo Scientific) and stored at −80 degrees. RNA purity was evaluated by the ratio of absorbance at 260/280 nm and RNA quality was evaluated through visualization of the 28S:18S ribosomal RNA ratio on a 1% agarose gel (Supplementary Figure 1A). Total RNA (1 μg) was synthesised into cDNA using Vilo cDNA synthesis kit (Invitrogen) and stored at −20 degrees.

### Real-time PCR

Real-time PCR was conducted using the ABI 7900 HT instrument (Applied Biosystems) following supplier instructions. Taqman^®^ Gene Expression Assay Kits (Applied Biosystems) were used to analyse the gene expression of the 9 coding PKCs genes. The ratio of stromal tissue to epithelium tissue in normal tissue and cancer tissue samples was analyzed using KRT18 and VIM taqman^®^ gene expression assays in 12 random tissue samples (Supplementary Figure 1B). A panel of nine housekeeping genes were evaluated using excel Normfinder and the four most stable genes were used for normalisation of each experiment (Supplementary Figure 1C).

### Immunohistochemistry

Tissue microarrays (TMA) were constructed from formalin fixed paraffin-embedded pre-treatment tumour samples using 1 mm cores, and they are in triplicate for each patient sample. Immunohistochemistry was performed using a Vectastain ABC Kit (Elite), as per the manufacturer's instructions. Slides were deparaffinised, rehydrated and heated-antigen retrieval was performed using Trilogy solution (Cell Marque Corporation). Endogenous peroxidase activity was blocked using hydrogen peroxide (3%) for 30 min and sections were blocked using goat serum and incubated with rabbit anti-human phospho-PKC Beta II (phospho S660) (Abcam, 1:300 dilution) for 1 h at room temperature. Sections were then incubated with secondary antibody for 30 min at room temperature. Diaminobenzidine (Sigma) was used to visualize staining and sections were counter-stained with haematoxylin, dehydrated and mounted. Stained sections were scanned using a scanscope XT digital scanner and ImageScope software (Aperio Technologies). Expression was assessed by scoring positive cell count and intensity in the epithelium and stroma by 2 reviewers who were blinded to the TRG status of the patient. Positivity was assessed using 7 categories (0%, 10%, 25%, 50%, 75%, 90% and 100%). Intensity was assessed using a scale of 0, 1, 2 and 3 which correlated with negative, weak, medium and strong staining, respectively. The % distribution and intensity values were multiplied to give the ‘H score’ for each tissue core.

### Cell culture and stable transfection

HCT 116 colorectal carcinoma cells were purchased from ATCC (ATCC^®^ CCL-247^™^ with certificates of analysis) and were maintained in Dulbecco's modified Eagle's medium (DMEM) (Sigma), supplemented with 10% (v/v) fetal calf serum, 10 mM L-Glu, and 5 mg/ml penicillin/streptomycin. HCT116 cells were transfected with pcDNA3/HA-PKC Beta II or empty pcDNA3 vector using LipofectAMINE Plus (Invitrogen). 24 h after transfection HCT116 cells were split into medium containing G418 (1.5 mg/ml) and maintained for 14 days with regular replenishment of medium and drug. Cells were examined for the expression of HA-PKC Beta II by western blotting. Cells overexpressing HA-PKC Beta II were maintained in Dulbecco's modified Eagle's medium supplemented with 1.5 mg/ml G418.

### Colony formation assay

Stable cell lines were harvested with trypsin/EDTA, washed with DMEM and counted using a haemocytometer. 500 cells were plated per well of a 6 well plate and incubated at 37°C in 5% CO_2_ for 10 days. Following this time, the cells were fixed in 96% ethanol for 10 min and subsequently stained with 0.05% crystal violet for 20 min. The wells were washed carefully and allowed to dry. Colonies were counted and recorded and a colony was deemed to be of 50 cells or more in size.

### 3-dimensional cell cultures

Individual wells of a 6 well plate were coated with Matrigel^™^ (BD Biosciences) and placed in an incubator at 37^°^C for 30 min. Stable cell lines were trypsinized and counted. 50,000 cells/ml were resuspended in DMEM supplemented with 2% Matrigel^™^. Cells were placed in Matrigel^™^ coated wells for 30 min at 37^°^C, after which DMEM supplemented with 2% Matrigel^™^ was added to the cultures. Cells were maintained in culture for 6 days in an incubator at 37^°^C, 5% CO_2_ with fresh medium added every 2 days and cultures imaged every 24 hours (Olympus cellSens Dimension 1.12). On day 6, cultures were harvested using EDTA/PBS, fixed with paraformaldehyde (PFA) and stained for confocal analysis (Zeiss LSM 710).

### Scratch wound assay

Adhesive wound assay inserts (Ibidi) were used to generate wounds in confluent layers in a 6 well plate. To do this, stable cells were trypsinized and seeded into the plate to form a monolayer. After overnight growth and attachment, the insert was carefully removed and the cells were starved of serum by adding serum free media for 4 hours, after which DMEM was added to the cells and they were imaged immediately (T0) using (Olympus cellSens Dimension 1.12). Cells were maintained in an incubator at 37^°^C, 5% CO_2_ for 24 hours, after which time images of the wound closure were taken as described above. The percentage wound closure was analyzed using Wimasis image analysis.

### Real time migration and invasion analysis

The rate of cell migration and invasion was monitored in real-time with the xCELLigence system CIM-plates. 4 hours prior to the experiment stable cell lines were serum starved. The upper chamber (UC) of the CIM-plates was coated with 1 ug/ul of fibronectin and for invasion experiments an additional 1:40 solution of Matrigel^™^ (BD Biosciences) was added. 30,000 cells were seeded in each well of the UC in serum free media. DMEM was added to each well of the lower chamber (LC). The CIM-plates was left in an incubator for 1 hour to allow cell attachment. The impedance value of each well was automatically monitored by the xCELLigence system (xCELLigence RTCA DP, ACEA) for duration of 72 hours and expressed as a cell index value (CI) value.

### Real time etoposide assay

The rate of cell death and proliferation was monitored in real time using the xCELLigence E-plate system. Either 30 000 HCT116 cells overexpressing PKC Beta II or 30 000 HCT116 cells expressing empty vector were placed in each well. The cells were allowed to adhere and proliferate for 16 hours following which time 15 μM of etoposide (Sigma) was added to the cells. The impedance value of each well was automatically monitored by the xCELLigence system for 32 hours and the data was normalised at the point the etoposide was added.

### IGF-1 and PMA treatment of cells

Cells were starved of serum for four hours prior to treatment with 50 ng of IGF-1 for the indicated times. The PKC inhibitor, Gö6983 (1 μM), was added 5 minutes prior to phorbol 12-myristate 13-acetate (PMA) and PMA (100 nM) was added 15 minutes prior to IGF-1. Cell lysates were prepared and run on 10–12% gradient gels and then transferred to membranes, which were blocked for 1 h at room temperature in TBS containing 0.05% Tween 20 (TBS-T) and 5% milk (w/v). All primary antibody incubations were overnight at 4°C. Secondary antibody incubations were carried out at room temperature.

### FRET imaging and analysis

HCT116 cells were rinsed and imaged in Hanks' balanced salt solution containing 1 mM Ca^2+^. Images were acquired and analysed as previously described [55]. For PKC activity measurements cells were co-transfected with CKAR and RFP tagged rat PKC Beta II. For AKT activity measurements cells were transfected with plasma membrane targeted BKAR [56]. Cells were starved of serum 4 hours prior to treatment of plates with IGF-1.

### Statistical analysis

Statistical analysis was performed using SPSS 20 Statistical Package unless stated otherwise. Significance between two groups was determined by the Mann Whitney *U* Test. Significance between more than two group was determined by the Welch Test and differences between these groups were determined by the Bonferroni Test. Disease Free Survival was analyzed using Kalpain Meir survival curves and the Log Rank Test, significance is reflective of univariate analysis. For matched patient samples differences in gene expression levels was determined for each individual patient using Pair Wise Fixed Reallocation Randomisation Test^©^ as per REST^©^ software. For all statistical analysis differences were considered to be statistically significant at *p* < 0.05.

## SUPPLEMENTARY MATERIALS FIGURES


