# Supplementary material for: Protein kinase C beta II suppresses colorectal cancer by regulating IGF-1 mediated cell survival
Source: Oncotarget. 2016 Mar 14;7(15):20919–33. doi: 10.18632/oncotarget.8062 (PMC4991501; doi:10.18632/oncotarget.8062)
Supplement: Supplementary file 1 [file oncotarget-07-20919-s001.pdf]

Protein kinase C beta II suppresses colorectal cancer by regulating IGF-1 mediated cell survival

Supplementary Materials

A

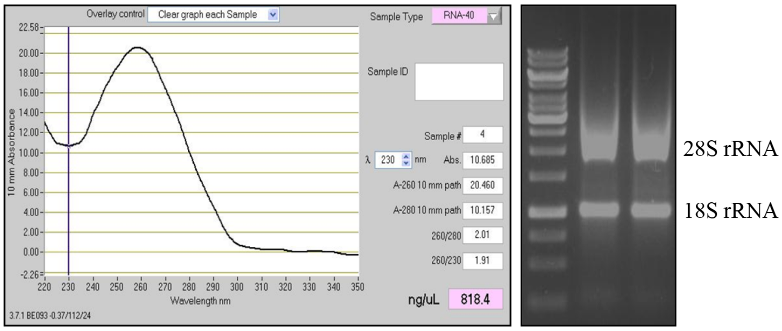

B

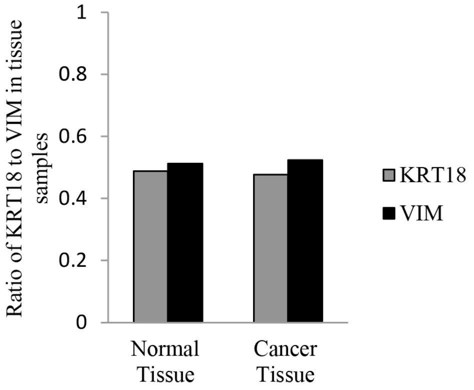

C

Gene name	Stability value	Standard error	Best gene
PGK1	0.152	0.140	PGK1
GUSB	0.307	0.148	GUSB
PP1A	0.412	0.203	PP1A
HRPT1	0.547	0.104	HRPT1
RPLPO	0.614	0.109	
B2M	0.929	0.099	
PMM1	1.004	0.138	
TBP	1.472	0.268	
ACTB	2.002	0.097	

Supplementary Figure S1: Validation of real time PCR. RNA purity and quality was analysed for all samples using the ratio of the absorbance at 260 and 280 nm. The RNA integrity was analysed for all samples by visualization of the 28S:18S ribosomal RNA ratio on a 1% agarose gel. The ratio of stromal tissue to epithelium tissue in normal tissue and cancer tissue samples was analysed using KRT18 (epithelium marker) and VIM (stromal marker) in 12 random tissue samples. To identify the best housekeeping genes to normalize the data a panel of 9 housekeeping genes were tested against tissue samples and analysed in excel using Normfinder. (**A**) Representative graph from Nanodrop Spectrophotometer displaying the quantity and purity of the RNA. Representative image of agarose gel displaying the 28S:18S ribosomal RNA ratio for a cancer and matched normal sample. (**B**) The ratio of epithelium to stromal tissue was analysed based on the expression of KRT18 and VIM in the normal and cancer tissue. (**C**) Table of results displaying the stability value of all 9 genes tested based on normfinder.

A

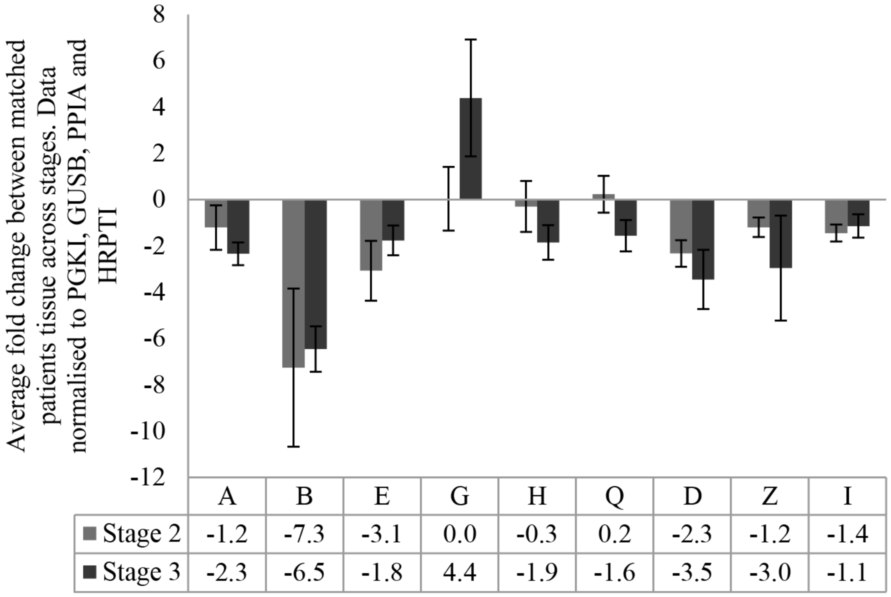

	A	B	E	G	H	Q	D	Z	I
Stage 2	-1.2	-7.3	-3.1	0.0	-0.3	0.2	-2.3	-1.2	-1.4
Stage 3	-2.3	-6.5	-1.8	4.4	-1.9	-1.6	-3.5	-3.0	-1.1

B

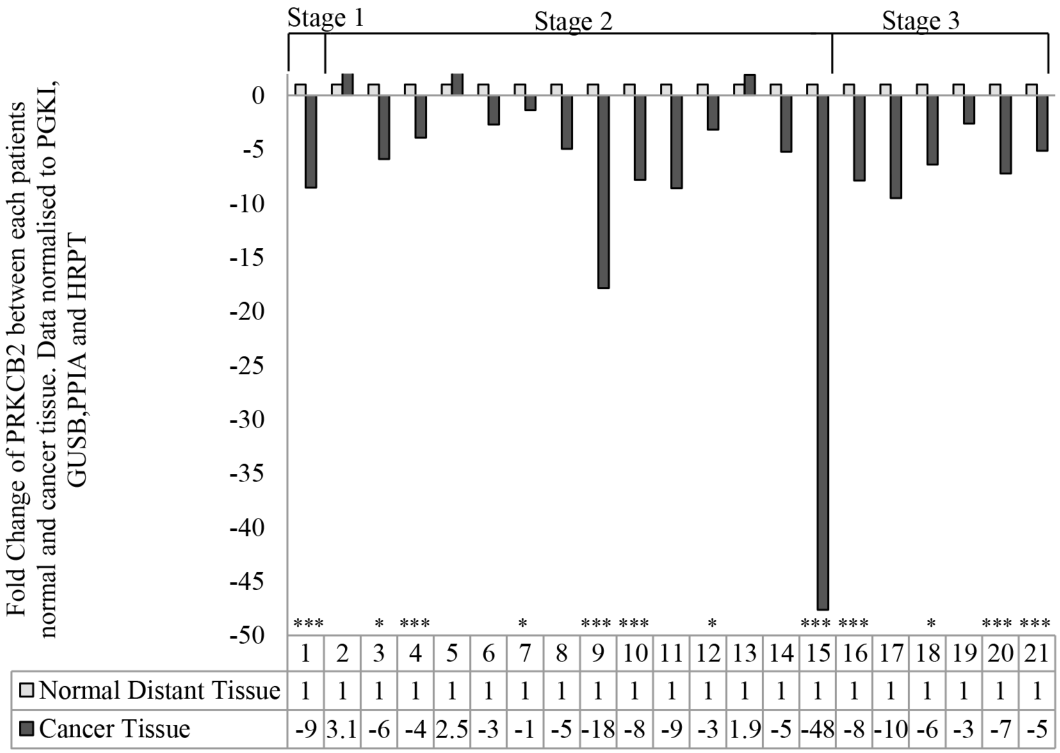

	1	2	3	4	5	6	7	8	9	10	11	12	13	14	15	16	17	18	19	20	21
Normal Distant Tissue	1	1	1	1	1	1	1	1	1	1	1	1	1	1	1	1	1	1	1	1	1
Cancer Tissue	-9	3.1	-6	-4	2.5	-3	-1	-5	-18	-8	-9	-3	1.9	-5	-48	-8	-10	-6	-3	-7	-5

Supplementary Figure S2: Gene expression analysis. (**A**) Average fold change of 9 PKC coding genes in cancer tissue of patients with stage 2 (n = 14) and stage 3 (n = 6) colon cancer. Each individual patients fold change was analysed using the REST© software (No statistical significance in expression between stages for any of the PKC coding genes as determined by paired student *T*-test). (**B**) Fold change of PRKCB in cancer tissue of each patient. Results were obtained by comparing mRNA level of PRKCB in individuals normal tissue compared to levels in that individuals cancer tissue (Statistical significance based on Pair Wise Fixed Reallocation Randomisation Test ©, *$p < 0.05$ and ***$p < 0.01$).

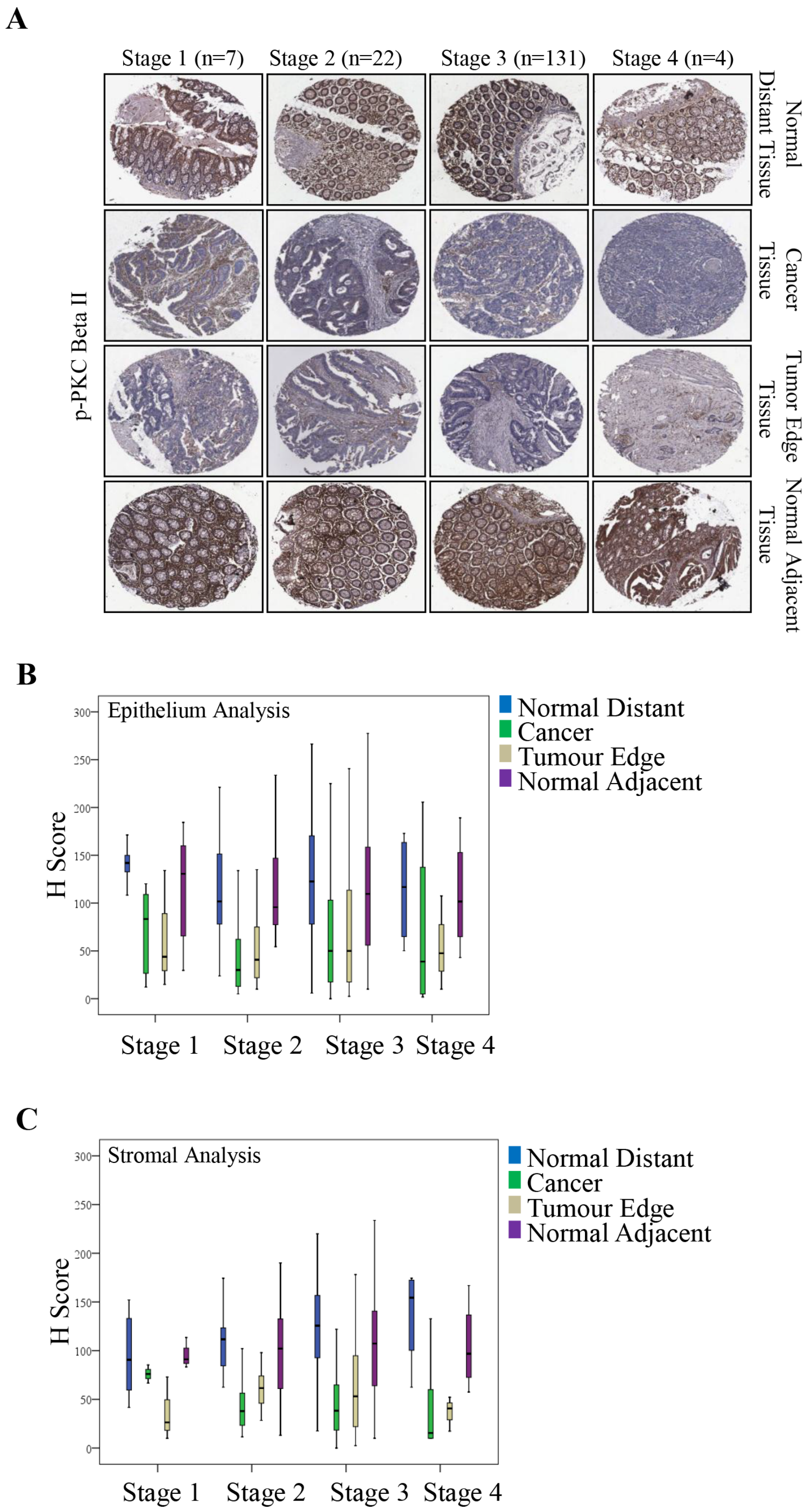

Supplementary Figure S3: Expression of PKC Beta II across different stages of CRC patients. PKC Beta II expression was assessed by immunohistochemistry in normal distant and cancer tissue samples in stages 1, 2, 3 and 4 of CRC patients. (**A**) Representative image of PKC Beta II staining for Normal Distant, Cancer tissue, Tumor Edge and Normal Adjacent tissue in stage 1 (n = 6, 7, 7, 3), stage 2 (n = 21, 22, 12, 16), stage 3 (n = 120, 131, 101, 101) and stage 4 (n = 4, 4, 3, 4) CRC patients. (**B**) Box plot representing the difference in PKC Beta II H score in Normal Distant, Cancer, Tumor Edge and Normal Adjacent epithelium tissue across stage 1, stage 2, stage 3 and stage 4 CRC patients (No statistical difference in expression between stages as determined by Welch test and Bonferroni test). (**C**) Box plot representing the difference in PKC Beta II H score in Normal Distant, Cancer, Tumor Edge and Normal Adjacent stromal tissue across stage 1, stage 2, stage 3 and stage 4 CRC patients (No statistical difference in expression between stages as determined by Welch test and Bonferroni test).

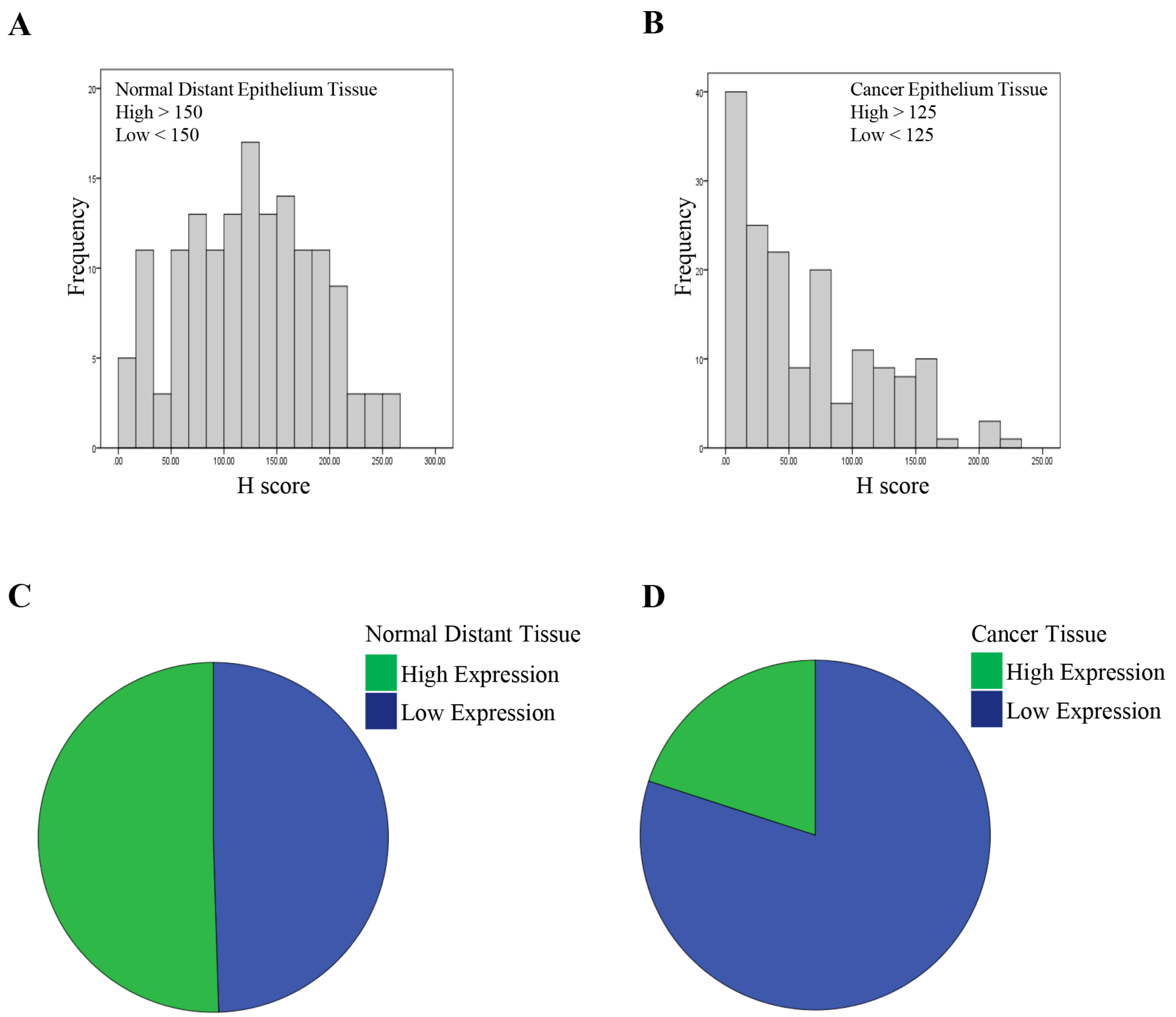

Supplementary Figure S4: Immunohistochemistry analysis. (**A** and **B**) Histograms used to determine the high and low expression values of PKC BetaII in (A) normal distant epithelium tissue and (B) cancer epithelium tissue. (**C** and **D**) Pie chart representing the % of cases with high and low expression in the normal distant (C) and cancer tissue (D).

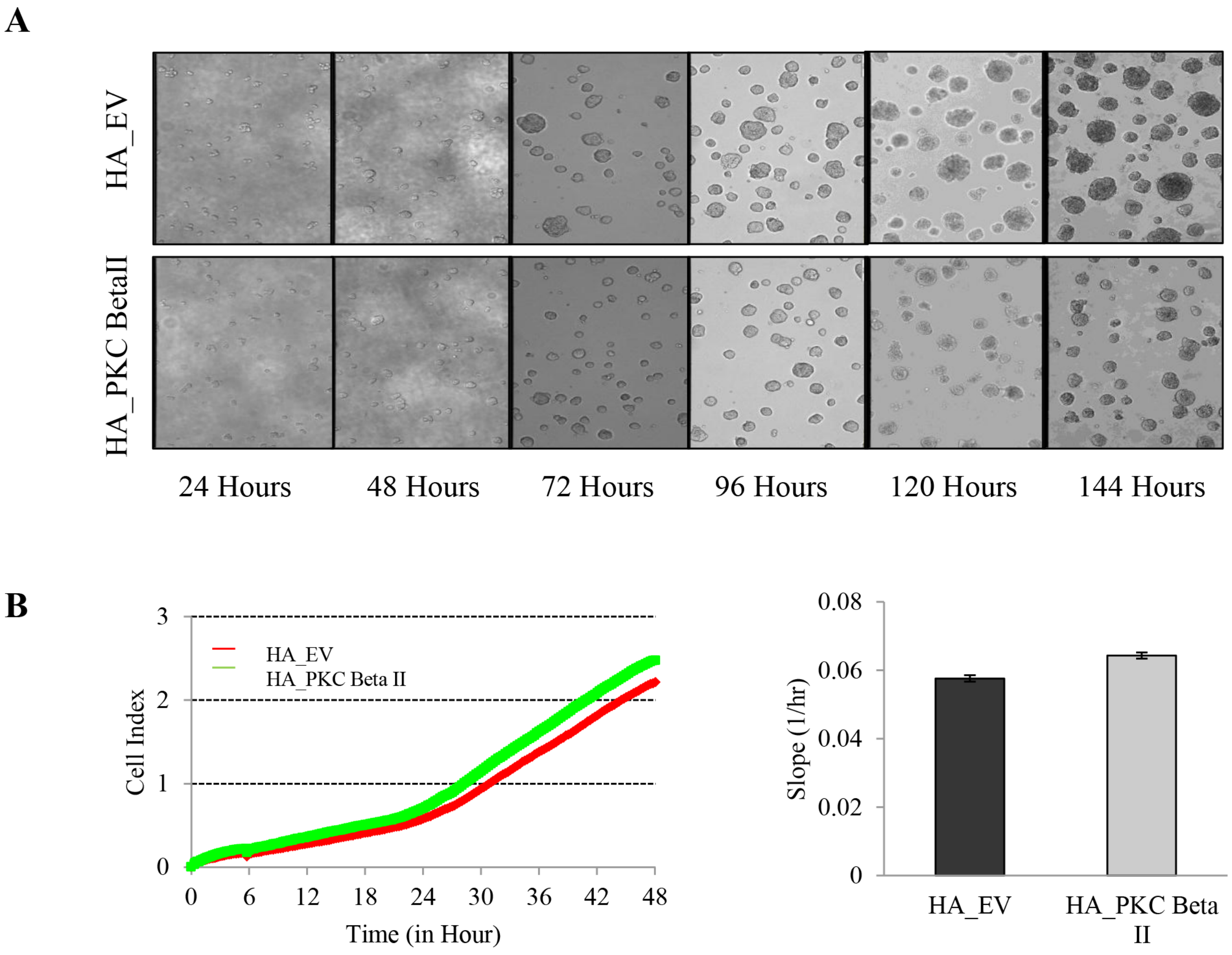

Supplementary Figure S5: Additional cell analysis. (**A**) Images demonstrating the difference in the size of cells growing in a 3D matrix at 24 hour intervals. (**B**) Representative graph of cells proliferating over a period of 48 hours analysed in real time on the xCELLigence system. Bar graph represents the rate of proliferation as determined by analysing the slope of the line between the 12 and 48 hour interval (no statistical difference found).
